# Urinary ^1^H-NMR Metabolomics in the First Week of Life Can Anticipate BPD Diagnosis

**DOI:** 10.1155/2018/7620671

**Published:** 2018-06-28

**Authors:** Maria Cristina Pintus, Milena Lussu, Angelica Dessì, Roberta Pintus, Antonio Noto, Valentina Masile, Maria Antonietta Marcialis, Melania Puddu, Vassilios Fanos, Luigi Atzori

**Affiliations:** ^1^Neonatal Intensive Care Unit, Neonatal Pathology and Neonatal Section, Azienda Ospedaliera Universitaria, University of Cagliari, SS 554, km 4.5 09042 Monserrato, Italy; ^2^Department of Biomedical Sciences, University of Cagliari, Cagliari, Italy

## Abstract

Despite the advancements in medical knowledge and technology, the etiopathogenesis of bronchopulmonary dysplasia (BPD) is not yet fully understood although oxidative stress seems to play a role, leading to a very demanding management of these patients by the neonatologist. In this context, metabolomics can be useful in understanding, diagnosing, and treating this illness since it is one of the newest omics science that analyzes the metabolome of an individual through the investigation of biological fluids such as urine and blood. In this study, 18 patients admitted to the Neonatal Intensive Care Unit of the Cagliari University Hospital were enrolled. Among them, 11 patients represented the control group and 7 patients subsequently developed BPD. A sample of urine was collected from each patient at 7 days of life and analyzed through ^1^H-NMR coupled with multivariate statistical analysis. The discriminant metabolites between the 2 groups noted were alanine, betaine, trimethylamine-N-oxide, lactate, and glycine. Utilizing metabolomics, it was possible to detect the urinary metabolomics fingerprint of neonates in the first week of life who subsequently developed BPD. Future studies are needed to confirm these promising results suggesting a possible role of microbiota and oxidative stress, and to apply this technology in clinical practice.

## 1. Introduction

Neonates, and especially those born prematurely, are particularly vulnerable to oxidative stress, as their levels of antioxidant enzymes are inadequate and unable to protect the rapidly growing tissues, including the developing lung, from oxidative injury. Bronchopulmonary dysplasia (BPD) is one of the main complications of prematurity included in the spectrum of “oxygen radical diseases of neonatology” [1]. Oxidative stress plays a key role in the pathogenesis of respiratory diseases of the preterm newborns, as a consequence of inadequate antioxidant capacities and increased presence of multiple oxidative stimuli, which is caused by preterm birth, intensive care therapy, infections, and inflammatory status [2].

BPD is the most common pulmonary complication in preterm newborns and it represents one of the principal causes of death in the extremely preterm neonates born before the 32nd week of gestation or weighing less than 1500 g at birth. BPD is a very complex pathology and it has been extensively reviewed in terms of physiopathology, diagnosis, treatment, and prevention in recent years [3].

In the last decade, the progress of perinatal medicine allowed a better definition of the etiopathogenesis and the features of BPD, improving the survival rate of this class of preterm newborns, although to date it is still extremely difficult to resolutely and effectively treat newborns suffering from this illness [4].

Thus, the reduction of BPD incidence represents a fundamental step to reduce the harmful consequences of this pathology and further reduce the incidence of other diseases that disrupt the pulmonary function and the neural development of the patients.

Therefore, being able to identify precociously the subjects at higher risk of BPD would allow for an improvement of the outcome of these little patients and would pave the way for individualized medicine [5].

Personalized medicine uses new medical technologies discovered in the last decade such as the “omics” sciences. Among them there is metabolomics, a multidisciplinary science that integrates several aspects derived from different branches (physics, chemistry, biology, and medicine).

Metabolomics is defined as “the quantitative measure of the multiparametric dynamic metabolic response of the living systems to physiopathological stimuli or genetics modifications” [6]. Thus, it is the science that studies the metabolome, namely, the complex system of metabolites that are the final products of the biochemical reactions, released in biological tissues and fluids. Studying the metabolites of an individual makes it possible to outline its biochemical phenotype [7–9], and in this sense, metabolomics is able to provide a high-resolution picture of what is happening inside an organism in a certain moment of its life. This provides a systematic study of the biochemical fingerprints resulting from cellular processes.

Moreover, it is also possible to accurately define a pathological state and perform a monitoring of the metabolic response of an organism to particular therapies, but one of the most interesting aspects is that it allows for the precocious identification of certain diseases even in the preclinical stage. A field where this appears extremely relevant is the neonatal period. In fact, metabolic profiling may provide information about the risk, prediction, diagnosis, and prognosis in a variety of diseases [10–13]. Therefore, metabolomics appears a relevant tool to provide useful information for the early identification and the management of complex pediatric disorders such as BPD. The search for a urinary metabolic profile distinguishing newborns who will develop BPD from preterm controls would allow the best management of the illness and its complications. We have already published a study analyzing urine at birth, in the development of BPD [14]. These data were confirmed by a study on the amniotic fluid [15]. Only in the present study, which focuses on urine samples collected at day seven of postnatal life, the potential influences after birth were investigated in the first week of life. The main objective of this study was to characterize the urinary metabolome of newborns affected by BPD using ^1^H-NMR spectroscopy in order to detect a metabolic fingerprint to be used as a predictive and diagnostic tool for this pathology. In this respect, attempts have been made to find a unique metabolic profile that would discriminate preterms developing BPD from those who do not, in order to identify as early as possible who among neonates will later develop the pathology. It is known that oxidative stress is important in the pathogenesis of various types of neonatal diseases (e.g., BPD, respiratory distress syndrome, retinopathy of prematurity, necrotizing enterocolitis, and congenital malformation). However, there is a need for more information regarding the prediction and diagnosis of oxidative stress-related conditions in neonates. There is a lack of knowledge on the individual evolution when conditions of susceptibility to oxidative stress damage are present as in the case of preterm infants predisposed to BPD.

## 2. Patients and Methods

### 2.1. Patients

This study included 18 preterm newborns admitted to the Neonatal Intensive Care Unit (NICU) of Cagliari University Hospital from 1st January 2012 to 30th September 2014. The gestational age was under 28 weeks and the birth weight under 1500 g. Parents were informed of the purpose of the study and their written consent was obtained. The patients were subsequently divided into 2 groups: the first group (12 controls) comprised newborns not affected by BPD and the second group (7 patients) consisted of newborns that developed BPD. The first case group (3 males, 9 females) had a mean gestational age of 27.9 ± 0.9 weeks and a mean birth weight of 1017 ± 200 g. The study group (4 males, 3 females) had a mean gestational age of 27 weeks and a mean birth weight of 784 ± 87 g (see Supplementary Material (available here) for maternal and neonatal demographic information). A sample of urine was collected from each newborn (approximately 1 mL), using a noninvasive method at the seventh day of life. As previously described [11], a cotton ball was inserted into the disposable diaper; then urine was aspirated through the use of a syringe and transferred into a sterile 2 mL vial. After collection, all the vials were immediately frozen after the addition of sodium azide 0.1% (*w*/*v*) and stored at −80°C until metabolomics analysis.

### 2.2. Reagents

All chemicals used in this study were of analytical grade. Deuterium oxide (purity 99.9% in D) and the internal standard trimethylsilylpropanoic acid (TSP) for NMR analysis were purchased from Sigma-Aldrich (Milano, Italy). Deuterated phosphate buffer (pH 7.4; 1.5 M) with 10% TSP was prepared and used for pH adjustment.

### 2.3. NMR Analysis

A total of 540 mL of the urine samples was mixed with 60 mL of aqueous phosphate buffer solution 1.5 M (pH 7.4). TSP in D_2_O was added to provide an internal reference for the chemical shifts (0 ppm). Samples were randomly analyzed as previously described [11]. Shortly, all ^1^H-NMR spectra were acquired on a 500 MHz Inova (Varian Inc., Palo Alto, CA) spectrometer equipped with a 5 mm triple resonance probe with *z*-axis pulsed field gradients. One-dimensional ^1^H-NMR spectra were collected at 27°C with a Presat 1D Noesy pulse sequence for water suppression. The relevant parameters used were calibrated and used as follows: a spectral width of 7997.6 Hz, acquisition time of 1.5 s, relaxation delay of 2 ms, 90° pulse of 10.7 ms, and number of scans of 128. The spectra processing was performed using MestReNova software (Version 7, Mestrelab Research S.L., Santiago de Compostela, Spain). The water resonance region, between 4.36 and 5.40 ppm, was excluded in order to eliminate the residual water signal, as well as that of urea, between 5.40 and 6.16. Subsequently, each spectrum was segmented into consecutive “buckets” of fixed size, 0.04 ppm, excluding the TSP resonance region, and the corresponding spectral areas were integrated. The final area included the region between 0.4 and 10 ppm to perform a matrix in which rows are samples, called observation, and columns are buckets, called variables. The generated matrix was normalized to the total sum of integrated areas fixed to 100 in order to minimize the effect of different concentrations of urine samples [16].

### 2.4. Statistical Analysis

The final dataset was imported into the SIMCA-P (version 14.0, Umetrics, Sweden) program and was Pareto scaled prior to analysis. Principal component analysis (PCA) and orthogonal partial least squares-discriminant analysis (OPLS-DA), unsupervised and supervised methods, respectively, were applied to the NMR dataset. PCA is a projection method used to obtain a general overview on the state of samples, highlighting possible clusters, trends, or outliers.

OPLS-DA is a classification method that maximizes the covariance between the measured data of the *X* variable (peak intensities in NMR spectra) and the response of the *Y* variable (class assignment) within the groups. The generated *R* ^2^ *Y* and *Q* ^2^ values described the predictive ability and the fitting reliability, respectively. The model was validated by a permutations test (*n* = 200). The permutation test was used to check the validity and the degree of over-fit for the model. The importance of the discriminating variables has been indicated as VIPs (variables of importance on the projection). Using the VIP list, the most important variables were translated and identified by means of the Chenomx NMR Suite software (version 7, Chenomx Inc., Edmonton, Canada) and the HMDB database [17].

## 3. Results

**Figure 1:**
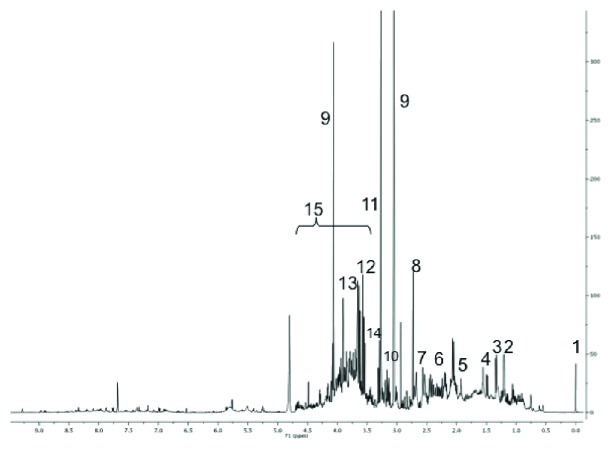
^1^H-NMR spectra of urine samples from a preterm infant with the following main assignments: 1 = TSP; 2 = 3-hydroxybutyrate; 3 = lactate; 4 = alanine; 5 = acetate; 6 = succinate; 7 = citrate; 8 = dimethylamine; 9 = creatinine; 10 = betaine; 11 = trimethylamine-N-oxide (TMAO); 12 = myoinositol; 13 = glycine; 14 = betaine; and 15 = glucose.

A representative spectrum of the ^1^H-NMR urine analysis is shown in Figure 1 pointing out the most relevant metabolites. The ^1^H-NMR spectroscopy coupled with multivariate statistical analysis was applied in order to identify possible metabolic profiles characteristic of preterm newborns who developed BPD compared to preterm newborns who did not develop BPD. The PCA of the dataset did not show the presence of any clusters (unpublished results). Based on Hotelling's *T* ^2^ test at 95% confidence and DModX test, no samples were identified as outliers. Therefore, OPLS-DA was applied in order to identify different possible metabolic profiles between the two classes.

**Figure 2:**
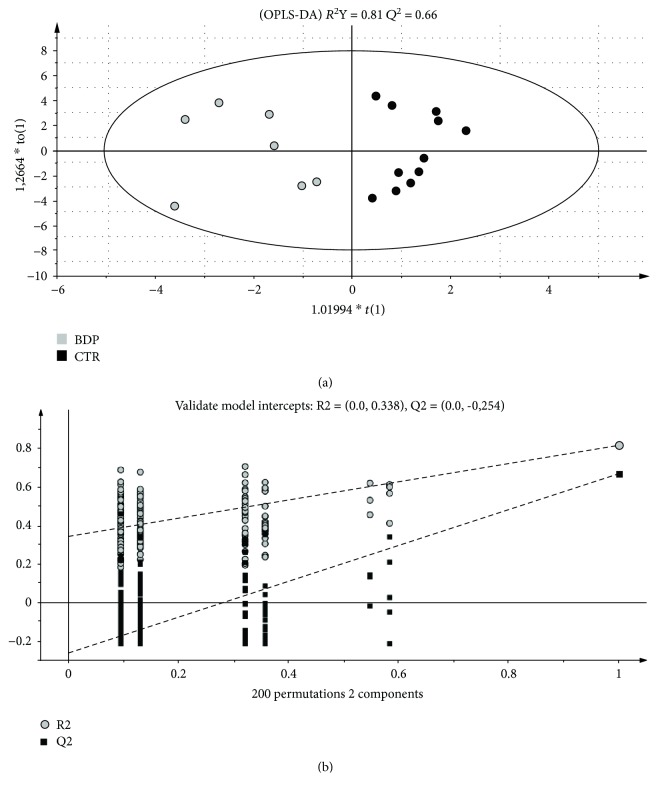
(a) OPLS-DA score plot of preterm controls (black circles) versus preterms who will develop BPD (grey circles) urine samples collected one week after birth. (b) The validation test of the model was assessed through 200 applications.

The OPLS-DA model is reported in Figure 2(a) (black dots: controls, grey dots: BPD). The results obtained showed that the samples were clearly separated into 2 groups indicating that controls and BPD-prone newborns presented a markedly distinct metabolic profile. The parameters of the model were *R* ^2^ *Y* = 0.81 and *Q* ^2^ = 0.66. By being both higher than 0.5, these parameters supported the goodness of the model. To validate the OPLS-DA model, a permutation test of the corresponding PLS-DA model was performed. The significance of the model was assessed through 200 applications in which all *Q* ^2^ values of permuted *Y* vectors were lower than the original one and the regression of the *Q* ^2^ line had an intercept below zero (Figure 2(b)). The results clearly indicated that the OPLS-DA model was statistically valid.

The metabolites that discriminated the two classes were identified by analyzing the S-plot and the VIP list (unpublished). Variables (metabolites) with a VIP score > 1 were considered and determined using the Chenomx NMR Suite 7.1. The main discriminant metabolites are identified and reported in Table 1. In detail, the metabolites contributing to the separation between controls and BPD were alanine and betaine (increased in the BPD group), trimethylamine-N-oxide (TMAO), and lactate and glycine (decreased in the BPD group).

## 4. Discussion

The main objective of our study was to characterize the urinary metabolome of newborns affected by BPD in order to identify a metabolomics fingerprint to be used as a predictive and diagnostic tool for this pathology. In our study population, the ^1^H-NMR analysis of the urine samples demonstrated that the preterms who go on to develop BPD show a different metabolic profile compared to the healthy controls.

In particular, the multivariate analysis of the urine spectra highlighted a cluster distribution, with a significant separation of the samples of the 2 groups of preterm neonates. In a previous work by Fanos et al. in 2014, the urinary metabolic profile in the first hours of life of extremely preterms who subsequently developed BPD was studied. In this study, it was hypothesized that the cellular metabolic activity associated with this condition could generate a distinctive model of metabolites, distinguishable from the metabolic profile of healthy newborns. Furthermore, it was also suggested that the destruction of the airways characterizing BPD could be the cause of the extensive cellular stress [14]. Even though there are some differences in the resulting metabolites, the main result of the present study confirms the hypotheses of the previous one.

Alanine is a nonessential amino acid made in the body from the conversion of the carbohydrate pyruvate or the breakdown of DNA and the dipeptides carnosine and anserine, which functions as a major energy resource. In skeletal muscle, the synthesis of alanine is directly proportional to the intracellular concentration of pyruvate that mainly increases when there is a high rate of degradation of fatty acid for energetic purposes, with a subsequent slowdown of the TCA cycle and ketone body formation. Pyruvate, in anaerobic conditions, cannot be oxidized in the Krebs cycle, so it is converted both into alanine and lactic acid. These compounds are released in the circulatory system, thus the plasmatic increase of alanine could explain its increase in urines. Furthermore, the glucose-alanine cycle is stimulated by the increase of plasmatic levels of glucocorticoids (cortisol), in response to stressful events of physic origin, as the BPD occurrence. Alanine levels parallel blood sugar levels. Alanine is an important participant and a regulator of glucose metabolism. It has been shown that intravenous glucose loading in infants with BPD resulted in a net increase in resting energy expenditure [18]. For this reason, the risk of pulmonary stress caused by an increase in basal oxygen consumption and carbon dioxide production resulting from glucose load has been suggested and our observed level of alanine might be linked to a deregulation of glucose and oxygen metabolism.

Lactic acid plays a role in several biochemical processes. Lactate is the end product of anaerobic glucose metabolism. The decrease in lactate urinary concentration in BPD patients could also be related to alterations in the Krebs cycle and Cory cycle. The study of Fanos et al. pointed out that during the first 24 hours of life, in newborns that will develop BPD, there is an increase in lactate level due to the activation of anaerobic glycolysis in response to the reduction of oxygen levels [14]. After 7 days of life, as demonstrated by the present study, urinary levels of lactate were decreased compared to those of controls, probably due to a compensatory response. In fact, the lactate at this stage can be transported to the liver and used as a gluconeogenetic precursor (Cory cycle). A decrease in lactate can be associated with increased oxidative stress. As lactate can have a protective effect by decreasing the formation of reactive oxygen species or the production of superoxide anion, the decrease in our patients can suggest the presence of an oxidative stress condition [19].

In our study, the concentration of betaine was increased in BPD patients. Betaine is a product of choline oxidation and a donor of methyl groups, and it is involved in numerous biological processes, in particular in the DNA methylation.

Methylation and sulfhydryl groups play a pivotal role in different cellular functions such as DNA control, signal transductions, and metabolic pathways. Oxidative stress has a significant impact on these mechanisms [20, 21]. Methyl donors are necessary for the metabolic pathway that produces S-adenosyl-methionine, which is the universal donor of methyl groups, thus it is essential for the methylation process of the DNA [22]. In the study performed by Wang et al., it has been shown that the oral ingestion of D9-betaine is related to the generation of circulating D9-TMAO regardless of the intestinal flora [23]. TMA, the precursor of TMAO, is not produced via intermediary metabolism; rather, it depends on intestinal microbial breakdown of choline and other precursors. Thus, the intestinal microbiota has an obligatory role in the formation of TMAO [24, 25] and plays a pivotal role in TMAO production [26]. For this reason, the decreased levels of TMAO in the urines of BPD patients suggest an alteration of the intestinal microbiota. Since we found this metabolite increased in our previous study at birth, it is plausible that microbiota have modified qualitatively in order to influence maturation processes.

Concerning glycine, its urinary levels in neonates affected by BPD was lower compared to those in controls. Glycine plays a key role in glutathione synthesis, which is a powerful antioxidant of free radicals and other oxygen reactive species. Several pathologies of the preterm, such as BPD, are related to oxidative stress with consequent low levels of glutathione [27]. Furthermore, glycine intervenes in the processes of detoxification of the organism, since it can conjugate with several harmful substances such as benzoic acid, to form nontoxic compounds eliminable in the urine, such as hippuric acid [28]. An antioxidant role of glycine has been suggested. Glycine ameliorated oxidative stress and the impairment in antioxidant enzyme activities, inhibited NF-*κ*B activation, and prevented expression of iNOS [29]. The observed decrease in glycine (and lactate) might indicate the presence of an oxidative stress condition or a lack of antioxidant defense.

High levels of hippuric acid were found in newborns suffering from recurrent respiratory tract infections (RRI), for which BPD is a predisposing factor. Bozzetto et al. in 2017 performed the metabolomics analysis of the urine samples of a children cohort suffering from RRI, showing that regarding the healthy controls there is an increase of hippuric acid. The latest is related to the specific composition of intestinal microbiota that seems to be altered in RRI children [30].

In addition to this, the microbiota of the airways is capable of modifying the normal development of the pulmonary immune system, giving rise to dysbiosis of the microbiota itself that predisposes to the occurrence of pulmonary pathologies such as BPD [31]. Differences in airway microbiota composition are also related to the different metabolic profiles of the exhaled breath condensate (EBC). It has been previously observed through metabolomic analysis that adolescents affected by BPD maintain in the EBC a metabolic profile different from the healthy controls [32].

The results of our study support all these hypotheses since the low urinary levels of glycine found in BPD newborns could be correlated with the necessity to not eliminate this amino acid through urine since it is fundamental in different biological processes such as those of detoxification and maintenance of microbiota homeostasis.

When we published our first study, we were concerned about the possibility to predict the outcome and the development of BPD at 6 weeks of life by an examination of urine samples at birth. In fact, the title contained a question mark. Our data suggested a genetic component in the determinism of BPD (already well-known in literature), associated to an intrauterine epigenetic component such as oxidative stress and inflammation [4]. The finding of Baraldi et al. in the amniotic fluid seems to support our hypothesis [15]. This study confirmed the involvement of some urinary metabolites already previously found (TMAO, lactate) indicating a possible role of microbiota that is realistically modified in the first week of life.

## 5. Conclusions

Our results identified a urinary metabolic fingerprint of the newborns suffering from BPD in the first week of life. The ^1^H-NMR metabolomics analysis of preterm newborns' urine established a trend of the metabolites in patients that will develop BPD when compared to healthy controls.

The results of this preliminary study, could pave the way to a potential prediction and early diagnosis of BPD.

Future studies should investigate the diagnostic value of a metabolomics approach to define the evolution of the preterm infants in relation to BPD and other oxidative stress-associated disorders.

## Figures and Tables

**Table 1:** Discriminant metabolites in urine collected one week after birth, between preterm controls and preterms who will develop BPD.

Metabolites	Chemical shift (ppm)	Trend in the BDP group
Alanine	1.48	↑
Betaine	3.25	↑
TMAO	3.27	↓
Lactate	1.34	↓
Glycine	3.57	↓

TMAO = trimethylamine-N-oxide.

## Data Availability

All data generated or analyzed during the study are included in this article.

## Supplementary Materials

Supplementary Materials Tables showing maternal and neonatal demographic information of the two groups of newborns.
