# Supplementary material for: Urinary 1H-NMR Metabolomics in the First Week of Life Can Anticipate BPD Diagnosis
Source: Oxid Med Cell Longev. 2018 Jun 28;2018:7620671. doi: 10.1155/2018/7620671 (PMC6046120; doi:10.1155/2018/7620671)
Supplement: Supplementary Materials — Tables showing maternal and neonatal demographic information of the two groups of newborns. [file 7620671.f1.docx]

| Group | Patients | Gender | Gestational age  (w) | Birth weight  (g) | Prophylactic corticosteroids | Mode of delivery | Apgar Score | | Early MV  (<4 hours of life) | Surfactant Treatment |
| --- | --- | --- | --- | --- | --- | --- | --- | --- | --- | --- |
|  |  |  |  |  |  |  | 1 min | 5 min |  |  |
| Controls | 1 | F | 28.9 | 830 | Yes | Emergency CS | 6 | 8 | No | Yes |
|  | 2 | F | 28.1 | 1130 | Yes | Emergency CS | 2 | 7 | Yes | Yes |
|  | 3 | M | 28.9 | 800 | No | Emergency CS | 3 | 5 | Yes | Yes |
|  | 4 | F | 27.7 | 1190 | Yes | Emergency  CS | 5 | 6 | No | Yes |
|  | 5 | M | 28.9 | 1060 | Yes | Emergency  CS | 3 | 4 | Yes | Yes |
|  | 6 | M | 27.9 | 1415 | Yes | Emergency CS | 6 | 6 | No | Yes |
|  | 7 | F | 28.9 | 1255 | Yes | Elective CS | 7 | 8 | No | Yes |
|  | 8 | F | 28.9 | 730 | Yes | Elective CS | 5 | 7 | No | No |
|  | 9 | F | 26.4 | 970 | Yes | VD | 5 | 7 | Yes | Yes |
|  | 10 | F | 27.1 | 915 | Yes | Emergency CS | 5 | 3 | Yes | Yes |
|  | 11 | F | 27.1 | 990 | Yes | Emergency CS | 8 | 9 | No | Yes |
|  | 12 | F | 27.1 | 870 | Yes | Emergency CS | 8 | 9 | No | Yes |
| BPD  patients | 1 | F | 26.3 | 740 | Yes | VD | 8 | 10 | Yes | Yes |
|  | 2 | M | 27.6 | 930 | Yes | Emergency  CS | 8 | MV | Yes | Yes |
|  | 3 | M | 27.6 | 830 | Yes | VD | 1 | 2 | Yes | Yes |
|  | 4 | F | 27.6 | 730 | Yes | VD | 1 | 3 | Yes | Yes |
|  | 5 | M | 27.0 | 807 | Yes | Emergency CS | 6 | 7 | Yes | Yes |
|  | 6 | M | 27.0 | 730 | Yes | Emergency  CS | 6 | 7 | Yes | Yes |
|  | 7 | F | 27.0 | 700 | Yes | VD | 2 | 4 | Yes | Yes |

Table 1. Demographic information of control group and BPD group of infants. MV: Mechanical Ventilation, CS: Cesarean Section, VD: vaginal delivery

| Group | Patients | Maternal characteristics |
| --- | --- | --- |
| Controls | 1 | Pregnancy induced hypertension; placental abruption; fetal distress |
|  | 2 | Suspected chorioamnionitis; fetal distress |
|  | 3 | Multiple pregnancy; intrauterine fetal death of a twin |
|  | 4 | - |
|  | 5 | Multiple pregnancy; intrauterine fetal death of a twin |
|  | 6 | Premature ruptures of membranes |
|  | 7 | Twin pregnancy |
|  | 8 | Twin pregnancy; intrauterine growth restriction |
|  | 9 | - |
|  | 10 | Multiple pregnancy; premature rupture of membranes |
|  | 11 | Multiple pregnancy |
|  | 12 | Multiple pregnancy |
| BPD patients | 1 | Intrauterine fetal death of a twin |
|  | 2 | Gestational diabetes mellitus; premature rupture of membranes |
|  | 3 | Twin pregnancy; premature rupture of membranes |
|  | 4 | Twin pregnancy; premature rupture of membranes |
|  | 5 | Twin pregnancy; premature rupture of membranes |
|  | 6 | Twin pregnancy; premature rupture of membranes |
|  | 7 | Twin pregnancy |

Table 2. Maternal demographic information of control group and BPD group of infants.
